# Effect of Digital applications on maternal as well as neonatal outcomes in Young pregnant girls: A Scope Review

**DOI:** 10.17533/udea.iee.v41n3e07

**Published:** 2023-10-24

**Authors:** Jasneet Kaur, Sheela Upendra, Shital Barde

**Affiliations:** 2 Professor, Ph.D. Email: sheelaupendra@scon.edu.in Symbiosis International University India sheelaupendra@scon.edu.in; 3 Associate Professor, Ph.D. Email: shitalbarde@scon.edu.in Symbiosis International University India shitalbarde@scon.edu.in; 4 Symbiosis College of Nursing, Symbiosis International (Deemed University), Pune, India. Symbiosis International University Symbiosis College of Nursing Symbiosis International (Deemed University) Pune India

**Keywords:** digital technology, adolescent mothers, infant, newborn, tecnología digital, madres adolescents, recién nacido, tecnologia digital, mães adolescentes, recém-nascido

## Abstract

**Objective.:**

To understand the effect of digital applications on maternal and neonatal outcomes in young pregnant girls.

**Methods.:**

A PubMed, CINAHL and Medline online database search was conducted, and related studies were included the databases were searched in order to carry out a more in detailed search of the available literature utilizing keywords like “digital technology”; “adolescent mothers”; and “infant, newborn”, as well as Boolean operators to generate papers pertinent which were correlating with the objective of the study.

**Results.:**

The findings revealed that the PPPs employed produced both positive and negative effects on mothers and newborns. Some were effective, especially in aspects related to improved mental health, while others did not necessarily support the adolescents in preparing for pregnancy and childbirth, but rather raised their anxiety levels. Similarly, the use of these apps decreased the use of emergency neonatal services by the adolescent mothers and the infants were lower in likelihood of exclusive breastfeeding. Participants appreciated the social media-based instruction, but this exposure did not translate into considerable change in routines and behaviors.

**Conclusion.:**

Digital and web-based solutions had the ability to influence adolescent pregnancy outcomes, but further research is needed to assess the extent to which these support services are useful in this Population Group.

## Resumen

**Objetivo.:**

Conocer el efecto de las aplicaciones digitales en los resultados maternos y neonatales en jóvenes embarazadas.

**Métodos.:**

Se realizó una estrategia de búsqueda en las bases de datos en línea PubMed, CINAHL y Medline utilizando los términos “digital technology”; “adolescent mothers”; y “infant, newborn”, y operadores booleanos.

**Resultados.:**

Los hallazgos revelaron que las APPs empleadas produjeron efectos tanto positivos como negativos sobre las madres y los neonatos. Algunas fueron efectivas, especialmente en los aspectos relacionados con la mejoría en la salud mental, mientras que otras nó ayudaron necesariamente a las adolescentes a prepararse para el embarazo y el parto, sino que más bien elevaron sus niveles de ansiedad. Del mismo modo, el uso de estas aplicaciones disminuyó la utilización de servicios neonatales de urgencia por las madres adolescentes y los neonatos tuvieron menor probabilidad de tener lactancia materna exclusiva. Las participantes apreciaron la instrucción basada en los medios sociales, pero esta exposición no se tradujo en un cambio considerable de rutinas y hábitos.

**Conclusión.:**

Las soluciones digitales y basadas en la web tuvieron la capacidad de influir en los resultados de los embarazos de adolescentes, pero se requiere de otras investigaciones para evaluar hasta qué punto son útiles estos servicios de apoyo en este grupo poblacional.

## Resumo

**Objetivo.:**

Conhecer o efeito dos aplicativos digitais nos resultados maternos e neonatais em gestantes jovens.

**Métodos.:**

Foi realizada uma estratégia de busca nas bases de dados online PubMed/Medline e CINAHL e utilizando os termos “tecnologia digital”; “mães adolescentes”; e “bebê, recém-nascido” e operadores booleanos.

**Resultados.:**

Os resultados revelaram que os APPs utilizados produziram efeitos positivos e negativos nas mães e nos neonatos. Alguns foram eficazes, especialmente em termos de melhoria da saúde mental, enquanto outros não ajudaram necessariamente os adolescentes a prepararem-se para a gravidez e o parto, mas antes aumentaram os seus níveis de ansiedade. Da mesma forma, o uso desses aplicativos diminuiu a utilização de serviços neonatais de emergência por mães adolescentes e os neonatos tiveram menor probabilidade de serem amamentados exclusivamente. Os participantes apreciaram a instrução baseada nas redes sociais, mas esta exposição não se traduziu em mudanças consideráveis ​​nas rotinas e hábitos.

**Conclusão.:**

As soluções digitais e baseadas na web tiveram a capacidade de influenciar os resultados da gravidez na adolescência, mas são necessárias mais pesquisas para avaliar até que ponto estes serviços de apoio são úteis neste grupo populacional.

## Introduction

Adolescent pregnancies are a global difficulty that affect excessive-, center-, and developing nations but, adolescent pregnancies are extra commonplace in marginalized groups round the world, regularly due to poverty and a loss of get right of entry too opportunities for schooling and employment.^1^ Adolescent females were negatively impacted by early motherhood, in addition to their spouses, households, groups, and colleges. Teenage mothers aren't prepared to become mothers; there need to be bodily, psychological, social, and cognitive instruction for the transition to motherhood.^2^ Teenage women find motherhood hard and complicated because they need to concurrently manage their responsibilities as mothers and the developmental demanding situations of childhood.^3^ They must adjust to their growing social responsibilities, the physical changes brought on by puberty, their substantial cognitive development, and the concern that comes with caring for a baby.^4^ The general public of adolescent mothers do no longer have sturdy socioeconomic backgrounds, making the adjustment to parenthood tough for them.^5^ The process of achieving the maternal role entails learning the necessary skills, developing appropriate behaviour, and establishing one's own maternal identity.^6^ Mother adjustment and the transition to adulthood are significantly impacted by preparation for taking on the mother role. 

The transition from being a teen without children to being a mother is challenging.^7^Teen mothers face a variety of physical, psychological, social, and spiritual challenges, including high-risk pregnancies and births, mental wellness issues,^8^^,^^9^ various kind of responsibilities, role conflict and identity uncertainty, insufficient social as well as spiritual hold, disturbance, and a lack of maternal skills when coping with novel circumstances and significant changes.^10^ Teen mothers and their neonates usually suffer from various kind of health risks because of the result of early pregnancies.^11^ The important reason of the mortality for girls in young age across the countries is complications during labor and childbirth, with low as well as middle income countries accounting for 99% of all maternal fatalities^12^^,^^13^ among women in the age from 15 years and 49 years internationally. Eclampsia,^14^ puerperal endometritis,^15^ and systemic infections^16^ are more prevalent in teenage moms between the ages of 10 years and 19 years than in those between 20 and 24.^17^ Additionally, between the ages of 15 and 19, more than 3 million insecure abortions occur each year, which raises maternal mortality, morbidity,^18^ and long term health difficulties.^19^ Preterm birth, gestational hypertension, low child birth weight, and other neonatal difficulties are among the prenatal and postnatal issues that these teenagers are more likely to experience.^20^ There is elevated risks for preterm birth, low birth weight, and neonatal death across all adolescent groups. Low Apgar scores at 5 minutes were more likely among babies delivered to adolescent moms who were 17 years old or younger.^21^

Family, friends, and partners are usually sources of support for those who are pregnant or just gave birth.^22^ Recent initiatives to help young mothers include home visits and community-based programmes reported that the deep ties developed during home visits may help home programmed visit generate greater results with teenagers who are harder to engage^23^ Social media is a significant component of digital media. It refers to internet-based channels of mass communication that enable user interactions, with the content being primarily user-generated.^24^^,^^25^ In this setting, pregnant women are increasingly turning to the internet for social and emotional support,^26^ as well as knowledge on pregnancy-related matters like diet. Expectant mothers may turn to social and web media or internet-based platforms rather than conventional sources as technology^27^ develops for information or support pertaining to pregnancy. Since the modern social structure has changed, many women are now emotionally and physically separated from their network of family and friends.^28^ Lack of knowledge and experience, the influence of peers, and high risk behaviors in teenagers; underscores the crucial role of health care professionals.^29^^,^^30^ Early motherhood is be viewed as one of significant public health concerns and is examined by obstetricians and gynecologists, pediatricians, child psychologists, sociologists, family doctors, and nurses.^19^ Providing high quality services involves awareness of the requirements of adolescent moms, their problems and talents. Like-minded women have the opportunity to interact with one another and gain social support through online alternative support networks.^31^ Web-based support services provide user anonymity, tumbling dishonor and encouraging the discussion of sensitive topics. They are reachable from anywhere at any time. Additionally, the majority of expecting moms utilize the internet to get information on varied topics, including labour and delivery as well as nursing, and they see it as a reliable source of knowledge. Therefore, the choices a mother makes about the care of her unborn child may be influenced by information she finds online.^32^

This Scope review is to essentially examine the existing facts that contributes to effects of the online or digital based Applications on maternal and the neonatal outcomes in pregnant adolescents’ girls. The research question focus on the following issues: (1) What are the various technological web applications used for maternal and neonatal well-being? and (2) What are the various maternal and neonatal outcomes in terms of benefits and effects of these technological applications?

## Methods

This is a scoping review. To find databases that could contain references, a variety of internet search engines were also employed. The review questions served as the direct inspiration for the specified criteria for choosing the studies. Written justifications were given for both inclusion and exclusion. Studies that focused on women from the age of 18 to 25 or identified their demographic as teenagers, early adolescents or adolescents were included. Any online service that allows users to share material with one another is considered a digital application. Physical (nutrition, exercise, breast-feeding behaviors, complications during labor, and risky habits like drinking alcohol and cigarette smoking) and psychosocial (mental wellness, anxiety, depression, feelings of isolation and tension, self-worth, birth preparation, and parenthood outcomes) factors have been assessed in relation to the outcomes of mothers. Preterm delivery, low birth weights, sudden infant death syndrome (SIDS), and obesity were all effects on children or infants. The requirements for inclusion were satisfied by all results gathered. We excluded reviews, abstract concepts, proceedings of conferences, the letters, commentary, comments, opinions, and book chapters in favor of studies with or without a comparison group that were pertinent to addressing our research concerns. We didn't include studies that weren't in English. Selected studies were put through a more thorough quality assessment using wide critical evaluation guidelines. PEO criteria was taken into account where the population of young mothers from 18 to 25 years of age was targeted who had exposure of any kind of technological application/web based application or digital application and displaying any kind of effect in terms of maternal and neonatal outcome. 

Strategies for data collection. The databases were selected for this investigation, and they were used for all phases of data collection. The CINAHL, Pubmed and Medline were all searched. The search was conducted using logical operators and keywords, to reduce data saturation. Therefore, it is crucial to show that a thorough, extensive, and wide search was conducted. MeSH words used for the search. Search strategy involved (("pregnancy in adolescence"[MeSH Terms] OR ("pregnancy"[MeSH Terms] AND "adolescent"[MeSH Terms])) OR (((((("adolescen*"[Title/Abstract] OR OR "young"[Title/Abstract]) OR ""teen*"[Title/Abstract]) OR "high school*"[Title/Abstract]) OR "girl*"[Title/Abstract]) AND ((("pregnan*"[Title/Abstract] OR "mother*"[Title/Abstract]) OR "birth"[Title/Abstract]) OR "maternal"[Title/Abstract]))) AND ("digital media"[MeSH Terms] OR ((((((((((((("social media"[Title/Abstract] OR "social network*"[Title/Abstract]) OR "social network site*"[Title/Abstract]) OR "forum*"[Title/Abstract]) OR "chatroom*"[Title/Abstract]) OR " communications media "[Title/Abstract]) OR "new digital media"[Title/Abstract]) OR "technology"[Title/Abstract]) OR "telehealth"[Title/Abstract]) OR "e-health"[Title/Abstract]) OR "m-health"[Title/Abstract)). Preferred Reporting Items for Systematic Reviews and Meta-analyses (PRISMA) criteria were followed.^33^ To guarantee research endorsed updated methods for providing for expectant mothers, we only included publications from the previous 12 years.

Study Selection. Two authors independently reviewed all papers that were found through database searches using MESH terms and worked with a third author to address disagreements. The entire texts of the studies chosen in level one were obtained, and the same two writers independently assessed each one to determine its eligibility. The grounds for exclusion were meticulously classified and recorded.

Data Extraction. Two reviewers independently collected the data from each report. The study design, Time period, participant characteristics, description of the intervention, maternal outcomes, newborn or child outcomes, findings, and limitations were all gathered using a standard proforma. Two reviewers collected data and worked independently.

Quality and Bias Assessment. The Newcastle-Ottawa Quality Assessment Scale^34^ quantitatively evaluates publications by assigning a s rating based on the selection, comparability, and exposure categories. The Cochrane Risk of Bias^35^ was used to analyse random controlled trials (RCTs), which were focusing on various aspects of trial design, conduct, and reporting. Joanna Briggs Institute instrument^36^ used to assess the qualitative and quasi experimental studies based on checklist. 

**Figure 1:**
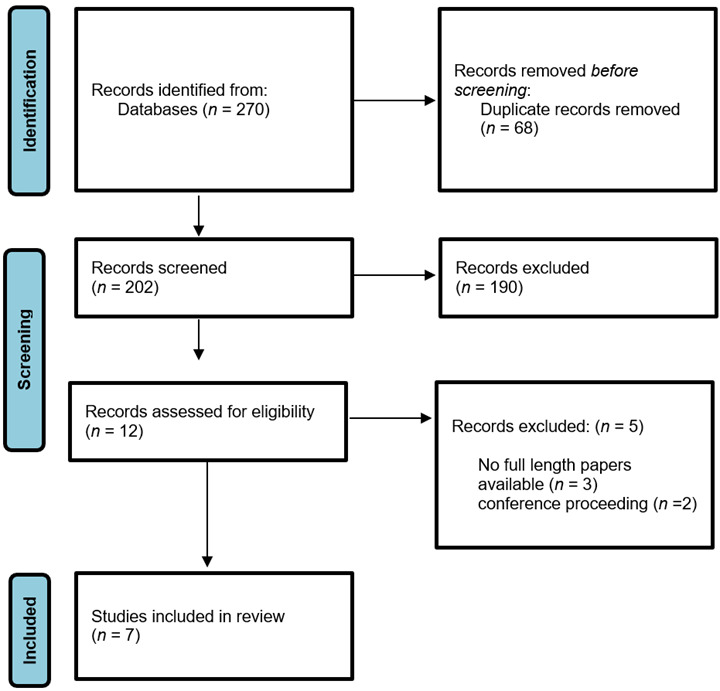
Prisma Flowchart

Search Results. A Boolean search for relevant phrases was performed yielded altogether 270 records. This restricted the number of records to 122 in CINAHL, 88 in Medline, and 70 in PubMed. Diagrams of PRISMA's flow were made as displayed in Fig 1. A few things were eliminated since they weren't pertinent to the subject of the study. After removing the duplicates, the abstracts of each publication were examined. 68 duplicates were removed and hence 202 records were found suitable and eligible for next screening. 2 independent authors performed the screening where 190 records were excluded with reasons (interventions were not based on online or digital platform *n*=92, age group more than 25 year*s n*=88, interventions was not related to desired outcomes *n*=10). Out of the left over 12 articles, 5 articles removed as no full length paper was available (*n*=3), and conference proceeding (*n*=2).

Synthesis of Results. The studies are summarised in Table 1. Adolescent girls in five studies received active intervention.^39^^-^^43^ Of these studies, one dealt with contacts between adolescents and medical professionals,^38^ two with interactions between adolescents^38^^,^^40^ and the final two dealt just with online material.^37^^,^^38^ Synthesis of results followed convergent synthesis where numerous outcomes gathered both before and after the intervention, and were assessed using self-reports, post intervention questionnaires and results targeting the research questions which specifically discussed in three sections which are the use of digital applications, Outcomes for the mothers and outcome for the neonates. 

## ResultsTable 1

**Table 1:** Study Characteristics

Author and Reference	Design	Sample size	Exposure	Key findings	Quality assessment Instrument	Rating
Fleming *et al*.^(37)^	Qualitative	19	Internet based Knowledge	This study showed that teenagers wanted and needed information on childbirth that was clear, accurate, and easily available. Giving the adolescent pregnant girls reliable electronic resources informed them, boost their confidence, and make them more prepared for giving birth.	JBI (qualitative research)	Include; risk of bias: low
Hudson et al.^(38)^	RCT	15 (Experiment Group) 19 (Control group)	Internet education resource	Assuming α=0.10,*p*<0.10; Intervention group had lower self-esteem than control group at 6 months; scale: RSEa Intervention group had higher levels of perceived competence after 6 months; scale: PPSb Intervention group had higher parenting satisfaction levels after 6 months; scale: WPBL-Rc ERd use reduced >50% in intervention group compared to control group (35.7% vs 70.6%); data collection: questionnaire Intervention group was less likely to exclusively breastfeed compared to control group; data collection: questionnaire	Cochrane Risk of Bias 2	Include; risk of bias: low
Jaideep Malhotra *et al.* ^(39)^	RCT (Longitudinal study )	512 Experimental group-255 Control group-257	Digital intervention on BCP	Stress levels and sleep habits have significantly improved	Cochrane Risk of Bias 2	Include; risk of bias: low
Vander *et al.* ^(40)^	Quasi experimental	22	Intervention by social media	Both teenage and adult low income pregnant females continue to have poor food quality.	JBI (Quasi experimental studies )	Include; risk of bias: low
Dhiren Modi *et al.* ^(41)^	Open Cluster RCT	11 PHC block on experimental and 11 in control group = 6493 mothers for intervention	Im TeCHO **-**Mobile technology	There were substantial improvements during antenatal period (adjusted effect size 15.7 [95% CI: 11.0, 20.4], *p*<0.001), postnatal period (adjusted effect size 6.4, [95% CI: 3.2, 9.6], *p*<0.001), early initiation of breastfeeding (adjusted effect size 7.8 [95% CI: 4.2, 11.4], *p*<0.001), and exclusive breastfeeding (adjusted effect size 13.4 [95% CI: 8.9, 17.9], *p*<0.001)	Cochrane Risk of Bias 2	Include; risk of bias: low
Logsdon *et al.* ^(42)^	Pretest Post test design	Experiment Group 151 Comparison group 138	web based app	The online intervention was effective in altering attitudes, perceptions of control, treatment intentions, and actual treatment receipt. No matter where they resided, teenagers' response to the intervention was the same, but the influence on views may depend on the dosage.	Newcastle-Ottawa Quality Assessment Scale	Include; risk of bias: low
Pontus Herricksson *et al.* ^(43)^	RCT	Intervention group 152 and control 153	Healthy Mom app	Three self-monitoring features (i.e., for weight-, diet- and physical activity) was associated with lower gestational weight gain (β = − 0.18,*p*= 0.043) and improved diet quality (β = 0.17,*p*=0.019). However, the number of APP sessions and page views were not associated with any of the outcomes	Cochrane Risk of Bias 2	Include; risk of bias: low

### Use of digital Applications

It is essential for teaching moms to offer reliable electronic links, mobile phone technologies, films, and access to provider and hospital websites, under the direction of their care providers.^37^ Hudson et al assessed the impact of Internet based intervention - a new mom network which could exchange experiences and learn from nurses how to take care of oneself and their newborns. MSNTV^TM^ was installed and linked to the Internet in the homes of the participants who were moms in the intervention group. Through their internet library and interactions with other moms and nurses, the New moms Network intervention offered parenting advice. Despite the fact that access devices are changing over time, the New Mothers Network website is well positioned for nursing-driven social support intervention over the Internet.^38^ iMumz maternity online programme where the infant care and parenting digital programme iMumz pregnancy has worked closely with expectant mothers to address these difficulties by providing a wide range of support and activities for maternal well-being in the convenience of their own homes. This app aims to assist expecting mothers in creating and maintaining a healthy, holistic lifestyle that starts before conception and lasts till after delivery. This software is a membership-based platform with a library of more than 800 doable activities in the categories of yoga, meditation, and specific exercises for bonding with the newborn. It is designed to help women maintain their physical and mental well-being.^39^ Vander et al used social media intervention (weekly prenatal health messaging).This social media intervention includes Health information which is distributed via Facebook (6 messages/week) and/or mobile text message (SMS; 6 messages/week) in the form of pregnant exercise, healthy recipes, nutrition, fun facts about pregnancy, and stress management.^40^

ImTeCHO is a mobile health intervention developed by Dhiren et al. to enhance the provision of maternity, neonatal, and child care services. The elements of the mobile phone application were home visit forms, a case details log, a work log, announcements, and an SMS information channel. During house visits, the Accredited Social Health activists (ASHA) fills out forms on her mobile device, which are then transmitted through the GPRS network to a server. Similar to this, the ANMs will receive a tablet to track high-risk cases and keep tabs on the ASHAs' performance.^41^ Web based depression interference on seeking therapy for depression was examined by Logsdon *et al*.^42^ The elements of the Internet-based depression intervention were Video Vignettes, Community Resources, and Common Questions and Their Answers. The webpage featured video vignettes of other teenage moms discussing their experiences with depression and how they were successful in finding therapy for it. Using data from the Healthy Moms app, Pontus Hanricksson *et al*.^43^ investigated the relationships among user engagement, with physical activity during pregnancy. The HealthyMoms app is a thorough 6-month programme that encourages a balanced diet and physical exercise in order to reduce excessive prenatal weight gain. Both Android and iOS devices may use the software. A text message with a link to a website that participants in the intervention group may view on their phone will be sent to them. Participants will be given instructions on how to register and download the app from Google Play or the App Store via the website. 

### Outcomes for the mothers

To understand the significance on self-preparation of mothers for giving birth in the hospital setting using electronic media, Fleming *et al*.^37^ did a research on perinatal education that calls for a thorough analysis. According to the research, exposure to electronic media did not necessarily help teenagers prepare for pregnancy and childbirth, but rather raised their anxiety levels. Despite the fact that many young moms had learned what to anticipate during childbirth, most of this information was fragmented, inconsistent, weakly connected, poorly referenced, not always helpful, and maybe even more confusing. Females may be better prepared to give birth with confidence when they enter the technological world of getting ready for giving birth at a hospital by taking care of moms' requirements. Similarly, the impacts of an Internet-based intervention are examined by Hudson et al. through the New Moms Network. The authors discovered that the intervention group's self-esteem scores on the Rosenberg Self-Esteem scale were considerably lower over the course of the 6-month period (*p*=0.04); however, they were unable to pinpoint a reason for this tendency. Despite the fact that access devices constantly changing, the New Mothers Network website is well positioned for nursing-driven social support intervention over the Internet and observed a good trend in social support levels after intervention, which was corroborated by the participants' qualitative remarks.^38^

However, in contrast Malhotra *et al.*^39^ displayed the different results through the reactions and pregnancies of women who participated in the iMumz maternity online programme during pregnancy. The research revealed a statistically significant reduction in stress levels and sleep habits. Additionally, it revealed that the incidence of preterm birth and low birth weight had decreased statistically significantly in the BCP (Baby care Program) trial group in comparison to the control group, and that the MFA between the mother and foetus had improved. 88% of patients reported much less stress after beginning BCP exercises on the app. The goal of Vandar et al. was to assess how well a social media intervention (weekly prenatal health messaging) affected food quality, as well as health beliefs and knowledge. Although participants were able to recognize items with added sugar and acknowledged the advantages of whole grains, their general understanding of the My Plate Guidelines was limited. Participants responded favorably to social media-based instruction, however there were minimal improvements in nutritional consumption and understanding. Social and web media seems to have the capacity to approach high risk women, but bigger research are required.^40^ ImTeCHO is a mobile health intervention developed by Dhiren *et al.*^41^ to enhance the provision of maternity, neonatal, and child care services. Government Accredited Social Health Activists (ASHA) and Primary Health care center (PHC) staff used the mobile and web-based ImTeCHO programme as a work tool, which increased the availability and calibre of MNCH services in difficult-to-reach locations.^41^ The impact of a web based depression interference on seeking therapy for depression was examined by Logsdon *et al*.^42^ Significant improvements in attitude, perception of control, desire to search for mental health therapy, and actual seeking for the treatment of depression were the result of the intervention. Untreated postpartum depression has a significant negative influence on a woman's connection with child her ability to perform at work and school, her desire to seek medical attention, her ability to be a good mother, and both her own and her kid's development. Increase treatment rates for depression via a low-cost Internet-based depression intervention. 

Using data from the HealthyMoms APP, Pontus Harricksson *et al*.^43^ investigated the relationships among user engagement, with physical activity during pregnancy. The connections between physical activity registrations and reduced gestational weight gain accounted for the majority of the results. But none of the outcomes were related to the volume of app sessions or page views. 

### Outcomes for the neonates

Hudson et al reported that Since 35.7% of mothers who received the intervention brought their child to the emergency room at least once, compared to 70.6% of mothers who did not (*p*=0.052), it was discovered that the use of emergency services for postpartum issues in the first six months had significantly decreased following the intervention. Each group had one suitable emergency department visit, one hospitalised infant, and one mother-infant pair treated for smoke inhalation. It was shown that adolescents in the intervention group were less likely to exclusively breastfeed than those who received standard care (*p*=0.06 [assume =0.10]).^38^ Vander *et al.*^40^ classified teenagers as being less likely than adults to breastfeed. Prenatal distress was linked to increased psychiatric risk, supporting the Developmental Origins of Health and Disease model. This finding of iMumz Maternity points to a "third pathway" for the transmission of disease within families beyond genetics and the postnatal effects of maternal psychopathology that affect fetal neurobehavioral development leads to better neonatal outcomes.^39^

## Discussion

The findings of this study highlight the current risk posed by uncontrolled pregnancy and childbirth websites and applications, as well as the rise of free wi-fi and free apps, which have implications for the entire globe owing to their accessibility on a global scale. As per the, information currently existing, the benefits and drawbacks of online media for teenagers who are pregnant or just delivered are still up for debate.^44^ Despite the fact that the maximum study's youthful participant grew up in a society where cutting-edge technology was often employed, this is still the case.^25^^-^^28^ In order to answer our research questions, we carefully looked at how teenagers' use of the internet affected different maternal and neonatal outcomes. A teen's life is greatly impacted by the online and digital media platforms and web-based technologies currently available and can have both good and bad results, especially when it comes to keeping up good friendships and refining unstable and potentially dangerous connections.^45^ Teens should carefully choose the internet networks they join in order to avoid psychological anguish in the future.^46^

Besides mental health outcomes, mothers have come up with the increased knowledge and confidence.^38^^,^^42^ Online platforms may be able to fulfil the unmet requirement for teen pregnant women to get active given the extensive use of online sources worldwide. The sole consistent finding in one of the research under consideration was mental health, with Logsdon et al. placing greater emphasis on depression than Hudson et al. As a result, it is impossible to draw conclusions about our second study topic.^47^ Few finding describe dietary improvements^40^ which is supported by other studies and are in consensus with that.^48^^,^^49^ The findings underscore the need for greater study in this vital area of regulating teenage pregnancies and the possibility of digitally solutions to reach teens who are pregnant or recently gave birth and feel more comfortable to search help online. 

The results indicated that majority of the teens believe on online and digital platforms. According to the findings of other various studies, the majority of pregnant women with higher education thought the health information they discovered on the Internet was trustworthy, dependable, and beneficial.^50^^-^^52^ Therefore, teenagers should carefully choose which online communities they join, according to the scientists, to minimize further emotional trauma. Given the widespread use of social media worldwide, social media platforms may potentially fulfil an unmet demand to involve young pregnant mothers. These sources make it likely for decision-makers and health professionals to provide this susceptible demographic with essential pregnancy-related information in an age-appropriate manner. Local or regional governments could be able to sway public health regulations via social media and internet platforms. By encouraging prenatal follow-up compliance and reducing pregnancy-related issues, it could prove to be a more efficient use of the funding that is available. Governments can expand the scope of their attention beyond prenatal care to encompass concerns like general women's health.

Limitations. The limits of the selected publications, as well as the lack of research in this vital field, are significant constraints of this systematic review, notably in nations, which are the most networked globally. Despite the extensive utilization of the web and social media by teenagers, few research has focused on how technology use among teenage mothers or young mother’s effects birth outcomes.

Conclusion. The study concluded that, there are vast digital applications in the form of internet-based applications, mobile technology applications, social media applications, and specifically designed applications like healthy Moms which has important implications not only for pregnant adolescents but their newborns also. On one side the digital platforms help adolescent mothers to reduce stress, sound sleep and active physical mobility but on other side it also raised anxiety level in pregnant adolescents. Similarly, by using these applications, the utilization of emergency neonatal services for the newborns of adolescent mothers has been decreased but neonates were less likely to have exclusively breastfeed as far as neonatal outcomes are concerned. Therefore, it evident that use of various digital applications which have both positive and negative effects on mothers and neonatal outcomes It is clear from the results that digital and web-based solutions have the ability to better the outcomes of adolescent pregnancies, but more thorough research is required to show how helpful these support services are. The study recommended that teens should take caution when selecting which online forums to join in order to reduce the risk of experiencing further emotional stress.
